# Comparison of interfascial plane injection and trigger point injection for upper trapezius myofascial pain syndrome in young women: a prospective cohort study

**DOI:** 10.3389/fmed.2026.1819707

**Published:** 2026-05-11

**Authors:** Ren Jiang, Yizhen Zhang, Hong Li, Wenwen Zhang, Xujie Ma

**Affiliations:** 1 Department of Anesthesiology, Ningbo Hospital of Integrated Traditional Chinese and Western Medicine, Ningbo, Zhejiang, China; 2 Department of Ultrasonography, Ningbo Hospital of Integrated Traditional Chinese and Western Medicine, Ningbo, Zhejiang, China

**Keywords:** interfascial plane injection, myofascial pain syndrome, pain management, shear-wave elastography, trigger point injection, upper trapezius muscle

## Abstract

**Background:**

Upper trapezius myofascial pain syndrome (UT-MPS) is a common painful disorder, particularly among young women. While both interfascial plane injection (IPI) and trigger point injection (TPI) are utilized, their comparative clinical profiles in this population are unclear.

**Materials and methods:**

This prospective cohort study compared the efficacy of TPI and IPI in 50 young women with UT-MPS. Although a non-randomized design was employed to reflect real-world clinical practice, blinded outcome assessment was maintained to minimize bias. Participants were assigned to either TPI (*n* = 25) or IPI (*n* = 25) and received three weekly ultrasound-guided injections of 0.2% ropivacaine. The primary outcome was pain intensity (NRS). Secondary outcomes included the Short-Form McGill Pain Questionnaire (SF-MPQ), Neck Disability Index (NDI), muscle stiffness assessed by shear-wave elastography (SWE), adverse events, and patient satisfaction, with follow-up to 12 weeks. Repeated measures analysis of covariance (RM-ANCOVA) was used for statistical analysis with baseline NRS as a covariate.

**Results:**

Both groups showed significant improvement over time in pain intensity, pain quality, functional disability, and muscle stiffness (all *p* < 0.05), with no significant between-group differences in clinical efficacy (all *p* > 0.05). However, the IPI group had significantly lower adverse event incidence (1.3% vs. 10.7%, *p* = 0.034) and higher patient satisfaction with treatment comfort and procedural pain tolerance (*p* = 0.033).

**Conclusion:**

For young female patients with UT-MPS, both ultrasound-guided IPI and TPI effectively relieved pain, improved cervical function and reduced muscle stiffness at 12 weeks. However, IPI offered distinct advantages in safety and patient satisfaction, which offers practical advantages in clinical settings where procedural simplicity is prioritized.

## 1 Introduction

Neck pain is a leading cause of disability globally, with myofascial pain syndrome (MPS) being a prevalent contributor (1). Data from the Global Burden of Disease (GBD) Study indicate that musculoskeletal disorders impose a substantial global disease burden, with neck pain ranking fourth. In 2017, the global prevalence of neck pain reached 288.7 million cases (2). Characterized by myofascial trigger points (MTrPs), MPS results in regional pain, stiffness, and functional limitation, significantly impairing quality of life (3). Prolonged poor posture, common among users of electronic devices, predisposes the upper trapezius—a key cervical stabilizer—to chronic strain and the development of upper trapezius myofascial pain syndrome (UT-MPS) (4). Non-invasive therapies such as stretching, topical analgesics and oral analgesics are standard treatments for UT-MPS (5), their benefits are often limited by delayed onset and dependence on patient adherence. Injection techniques offer a faster, more targeted alternative by delivering local anesthetics directly to pathological sites to inactivate MTrPs and restore fascial function (6).

When non-invasive therapies fail to provide adequate pain relief, TPI offers an effective targeted option. However, its efficacy can be inconsistent, partly due to the technical demands of precisely localizing MTrPs under high-resolution ultrasound and the procedural pain from multiple injections (7). Multiple studies have validated that TPI significantly alleviates pain and functional impairment in patients with MPS (8, 9). However, some studies have reported that pain relief after TPI is suboptimal in some patients, with a subset exhibiting no significant clinical benefit. This may be attributed to the heavy reliance on high-resolution ultrasound equipment for the precise localization of MTrPs. Furthermore, TPI often requires the simultaneous treatment of multiple MTrPs. Young females with UT-MPS often report higher pain scores and lower pressure pain thresholds, and the procedural discomfort can lead to reduced compliance or even refusal of subsequent sessions. These limitations led us to optimize treatment protocols, balancing therapeutic efficacy and patient experience.

Interfascial plane injection (IPI) presents an alternative approach. By depositing medication within defined fascial spaces under ultrasound guidance, IPI aims to release fascial adhesions and restore myofascial mobility, thereby breaking the “pain-tension-spasm” cycle (10). Compared with TPI, IPI requires less operator expertise and lower-end ultrasound equipment, as it targets consistent anatomical planes rather than variable MTrPs (11). A single injection can cover a broader area, potentially enhancing patient comfort (12). Despite growing evidence supporting IPI in chronic pain, no prospective study has systematically compared its efficacy, safety, and patient satisfaction against TPI specifically in young women with UT-MPS (13), a demographic notable for its high disease burden, elevated disability rates, and increased pain sensitivity (14).

Therefore, this prospective cohort study compared IPI and TPI for efficacy, safety, and patient satisfaction in this population. We hypothesized that IPI would provide comparable analgesic efficacy to TPI but with superior procedural comfort and fewer adverse events. Both techniques represent established ultrasound-guided procedures routinely performed in clinical practice, and this comparative effectiveness evaluation aimed to inform real-world clinical decision-making rather than test experimental interventions. Given the practical challenges of randomized allocation in this sensitive clinical demographic, we adopted a pragmatic prospective cohort design to evaluate the comparative effectiveness of IPI and TPI under real-world conditions.

## 2 Methods

A prospective cohort study was conducted. The study protocol was approved by the Ethics Committee of the Ningbo Hospital of Integrated Traditional Chinese and Western Medicine (formerly Ningbo Yinzhou District No.2 Hospital) (approval no.: 2023-053; Approval Date: January 4, 2024), and written informed consent was obtained from all participants after adequate explanation. As both interfascial plane injection and trigger point injection are established ultrasound-guided procedures routinely performed in clinical practice, formal clinical trial registration was not required per institutional policy for observational studies of standard therapeutic techniques. This study was conducted in the Department of Pain Medicine from June 2024 to March 2025, and a total of 50 young female patients who met the diagnostic criteria for UT-MPS were enrolled.

### 2.1 Inclusion and exclusion criteria

Eligible participants were young women aged 18 to 44 years with a body mass index (BMI) between 18.5 and 24 kg/m^2^. All patients fulfilled the diagnostic criteria for UT-MPS: primary unilateral cervicoscapular pain lasting at least 3 months, a baseline Numeric Rating Scale (NRS) score ≥ 3, and a Neck Disability Index (NDI) score > 8. Palpation had to reveal at least one active myofascial trigger point (MTrP) in the upper trapezius muscle, with reproducible referred pain upon compression. Patients were excluded if they had received physical therapies such as acupuncture or small needle-knife treatment within the preceding 3 months, or glucocorticoid therapy within the past month. Additional exclusion criteria included cervical disc herniation; concomitant cardiovascular, cerebrovascular, neurological, or autoimmune diseases; and inability to adhere to the treatment and follow-up schedule.

### 2.2 Patient enrollment and bias management

Participants were assigned to either the TPI or IPI group based on a pragmatic clinical approach that considered both physician expertise and patient preference. While we acknowledge that this non-randomized allocation may introduce selection bias, this design was chosen to better reflect real-world clinical practice and enhance patient treatment compliance, which is particularly crucial in a young female demographic with high pain sensitivity. To mitigate potential confounding, strict inclusion and exclusion criteria were applied, and outcome assessors remained blinded to the treatment allocation throughout the study. Furthermore, baseline clinical characteristics, including age, BMI, and baseline pain intensity, were monitored to ensure comparability between the two cohorts.

### 2.3 Blinding

Due to the distinct procedural nature of the injections, treating physicians and patients could not be blinded. However, strict blinding was maintained for outcome assessors and statisticians. To ensure the integrity of the blind, outcome assessments were conducted in a dedicated room separate from the treatment area by independent staff who were not involved in the injection procedures and remained unaware of group assignments. Patients were explicitly instructed at each visit not to discuss their specific treatment experience with the assessors. Furthermore, the skin of the injection site was inspected by a non-blinded nurse prior to assessment to ensure no identifying marks (e.g., localized bruising) would bias the assessor. Statisticians analyzed the data using group coding (Group A vs. B) and were only unblinded after the final statistical analysis was locked.

### 2.4 Intervention

**Figure 1:**
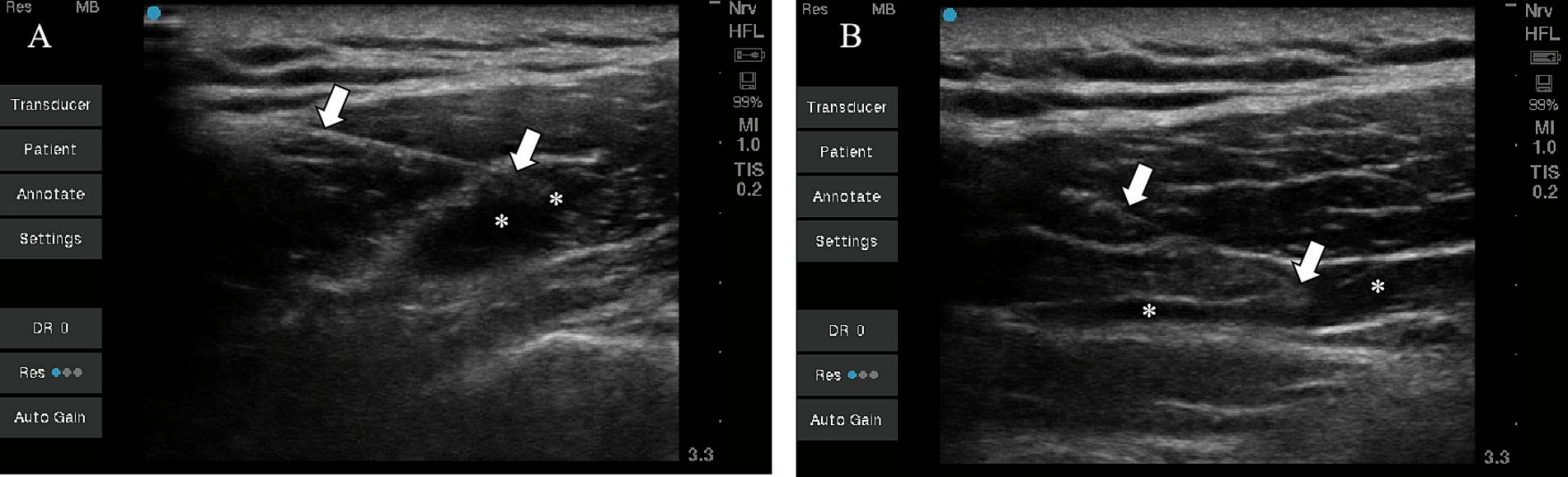
Ultrasound images of TPI and IPI procedures. Panel **A** shows TPI, where the hypoechoic shadow marked with white asterisks indicates ropivacaine accumulation around the MTrP. Panel **B** shows IPI, where the hypoechoic shadow marked with white asterisks indicates ropivacaine diffusion within the fascial space. White arrows indicate a 22-gauge, 6-cm needle. TPI, trigger point injection; IPI, interfascial plane injection; MTrP, myofascial trigger point.

A single physician with over 5 years of interventional pain management experience performed all procedures under real-time ultrasound guidance (Sonosite Edge, 12-MHz linear array transducer; imaging depth 3.3 cm). A 22-gauge, 6-cm needle was used with an in-plane approach, with patients seated and the neck-shoulder region exposed. In the TPI group (*n* = 25), 3 active myofascial trigger points (MTrPs) were first identified via systematic palpation combined with ultrasound (prioritizing the most tender points) and marked; after eliciting a local twitch response via quick in-and-out needle manipulation, a total volume of 20 mL of 0.2% ropivacaine was administered (6–7 mL per point, 20 s per point). Sonography showed hypoechoic accumulation around the MTrP. In the IPI group (*n* = 25), the point of maximal tenderness was selected and marked, the needle was advanced into the deep fascial plane (trapezius plane) beneath the marker, and after negative aspiration, a single bolus of 20 mL of 0.2% ropivacaine was slowly injected (60 s). Sonography showed hypoechoic distribution within the fascial plane. Both groups received three weekly treatments (Figure 1).

### 2.5 Outcome measure assessment

All assessments were conducted in a dedicated treatment room by physicians blinded to group allocation. Follow-up assessments were conducted at 1 week (7 days), 4 weeks, and 12 weeks after the initial (and only) injection session. Patients were evaluated using the Numeric Rating Scale (NRS) to rate pain intensity on a scale from 0 to 10 (15), which represented the average pain intensity experienced during daily activities over the previous 24 h, the Short-Form McGill Pain Questionnaire (SF-MPQ) to assess the sensory and affective dimensions of pain (16), and the Neck Disability Index (NDI) to quantify pain intensity and functional status (17). A trained ultrasonographer performed shear-wave elastography (SWE) of the upper trapezius at baseline and 12 weeks post-treatment using a Mindray Resona R9G ultrasound diagnostic system with an L15-3WU linear array transducer. The primary objective of SWE was to provide a quantitative and objective assessment of muscle stiffness (Young’s modulus), serving as a biomechanical correlate to subjective pain intensity (18). During the examination, patients were asked to keep their muscles in a natural relaxed state, and the transducer was placed parallel to the fiber direction of the trapezius muscle, with the imaging depth set at 4 cm. Only images with high stability and reliability coefficients were selected for subsequent analysis. The SWE mean (SWE_mean) and maximum (SWE_max) stiffness values (kPa) were measured within the muscle layer; three consecutive measurements were obtained at the same site, and the average values were calculated.

Adverse events were recorded after each injection session. Patient satisfaction was assessed using a self-designed questionnaire at the 12-week follow-up, which has been previously validated for interventional pain procedures. The questionnaire evaluated four dimensions: treatment comfort, pain tolerance, efficacy satisfaction, and overall satisfaction. Scores ranged from 0 (extremely dissatisfied) to 5 (extremely satisfied).

### 2.6 Sample size calculation

The sample size was determined based on the Numeric Rating Scale (NRS) as the primary outcome measure. To detect a minimum clinically important difference (MCID) of 1.5 points in NRS scores between the TPI and IPI groups, with an assumed pooled standard deviation of 1.5 and a two-sided *α*-level of 0.05, a sample of 18 participants per group was required to achieve a statistical power (1-*β*) of 80%. Considering a potential dropout rate of approximately 20%, we targeted a total enrollment of 50 participants (25 per group). Ultimately, all 50 enrolled participants completed the 12-week follow-up, ensuring that the study was adequately powered to evaluate the primary comparative objective (19).

### 2.7 Statistical analysis

All statistical analyses were performed using SPSS 20.0 (IBM Corp., Armonk, NY, United States). Continuous variables were expressed as mean ± standard deviation (SD) and evaluated for normality using the Shapiro–Wilk test and for homogeneity of variance using Levene’s test. For repeated measures data (NRS, SF-MPQ, NDI, and SWE values), two-way repeated measures analysis of covariance (RM-ANCOVA) was used to evaluate the main effect of time, main effect of group, and time-by-group interaction effect. Greenhouse–Geisser correction was applied when the assumption of sphericity was violated. Given the non-randomized design, baseline NRS scores were included as a covariate to adjust for potential confounding. Post-hoc between-group comparisons at each time point were performed using independent samples t-tests with Bonferroni correction for multiple comparisons. To assess the precision and clinical significance of the treatment effects, 95% confidence intervals (CIs) were calculated for the mean changes from baseline to each follow-up time point. Categorical variables, including the incidence of adverse events, were analyzed using Fisher’s exact test and reported with their corresponding 95% CIs. A two-sided *p*-value < 0.05 was considered statistically significant. Sensitivity analyses excluding potential outliers were performed to confirm the robustness of the primary findings.

## 3 Results

### 3.1 Patient characteristics and flow

**Figure 2:**
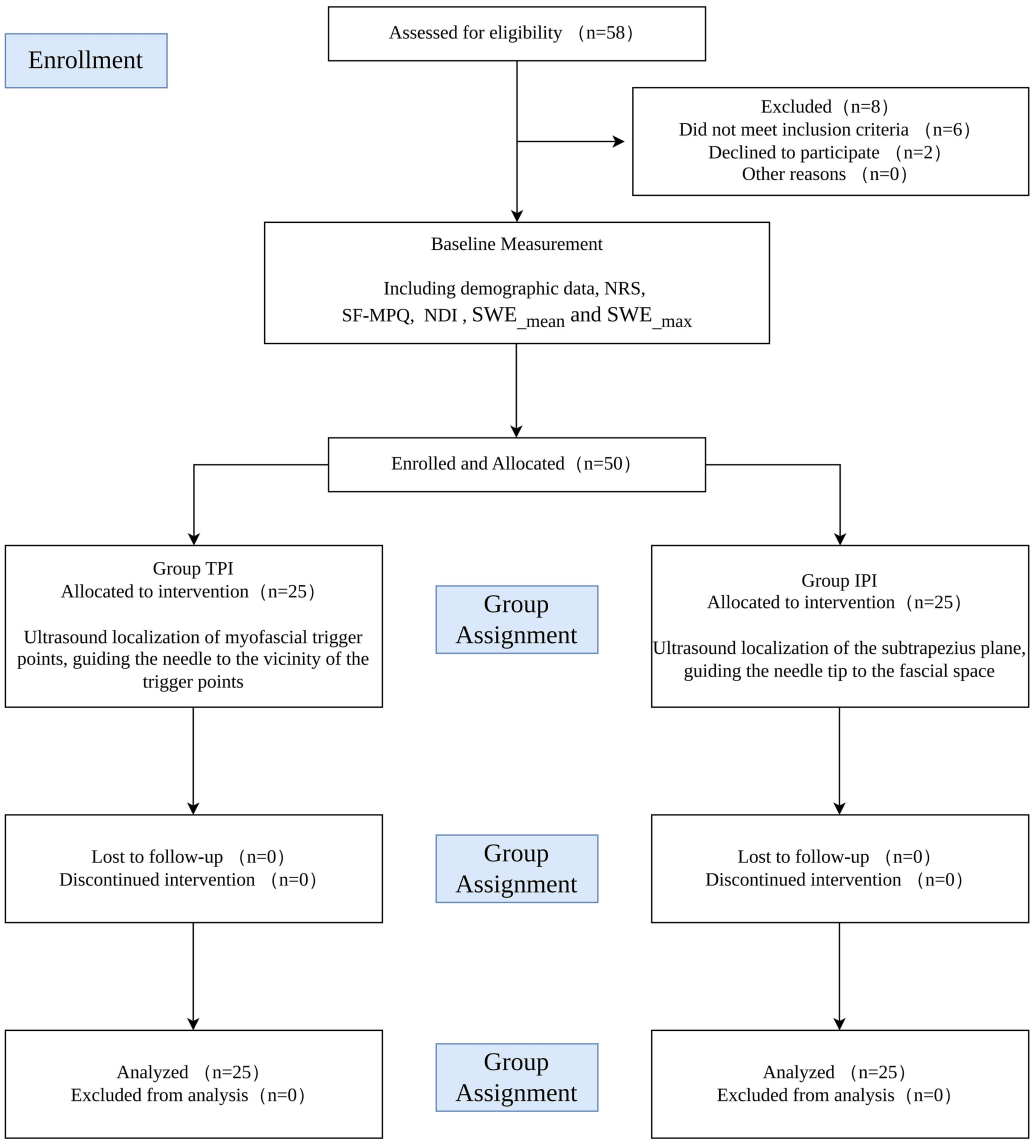
Study design and participant flow diagram. A total of 58 young female patients with upper trapezius myofascial pain syndrome (UT-MPS) were assessed for eligibility; 8 were excluded or declined participation, and 50 were enrolled and assigned to the TPI group (*n* = 25) or IPI group (*n* = 25). All 50 patients completed the 12-week study protocol without dropout. TPI, trigger point injection; IPI, interfascial plane injection. NRS, Numeric Rating Scale; SF-MPQ, Short-Form McGill Pain Questionnaire; NDI, Neck Disability Index; SWE, shear-wave elastography.

A total of 58 young female patients with UT-MPS (mean age 33.5 ± 5.3 years) were assessed for eligibility. Six were excluded due to painful comorbidities that could interfere with outcome assessment, and two declined to participate for personal reasons. The remaining 50 patients were enrolled and assigned to receive either TPI (*n* = 25) or IPI (*n* = 25). All enrolled patients completed the study protocol without dropout. Baseline demographic and clinical characteristics were well-balanced between the two groups, with no statistically significant differences observed in all indicators (all *p* > 0.05, Table 1). The detailed participant enrollment flow is illustrated in Figure 2.

**Table 1:** Baseline demographic and clinical characteristics of patients in TPI and IPI groups.

Characteristic	TPI group	IPI group	*p*-value
Age (years)	34.5 ± 5.6	33.8 ± 4.7	0.643
Height (cm)	160.2 ± 4.9	159.9 ± 5.0	0.865
Weight (kg)	53.6 ± 4.1	51.9 ± 4.5	0.181
BMI (kg/m^2^)	20.9 ± 1.9	20.1 ± 1.9	0.157
affected side (left/right)	11/14	9/16	0.773
Baseline NRS	3.88 ± 0.9	4.04 ± 1.1	0.573

TPI, trigger point injection; IPI, interfascial plane injection; BMI, body mass index; cm, centimeter; kg, kilogram. Data are presented as mean ± SD. *p*-values were derived from independent samples t-tests for continuous variables and Fisher’s exact test for the categorical variable (affected side) (all *p* > 0.05).

### 3.2 Pain intensity

**Figure 3:**
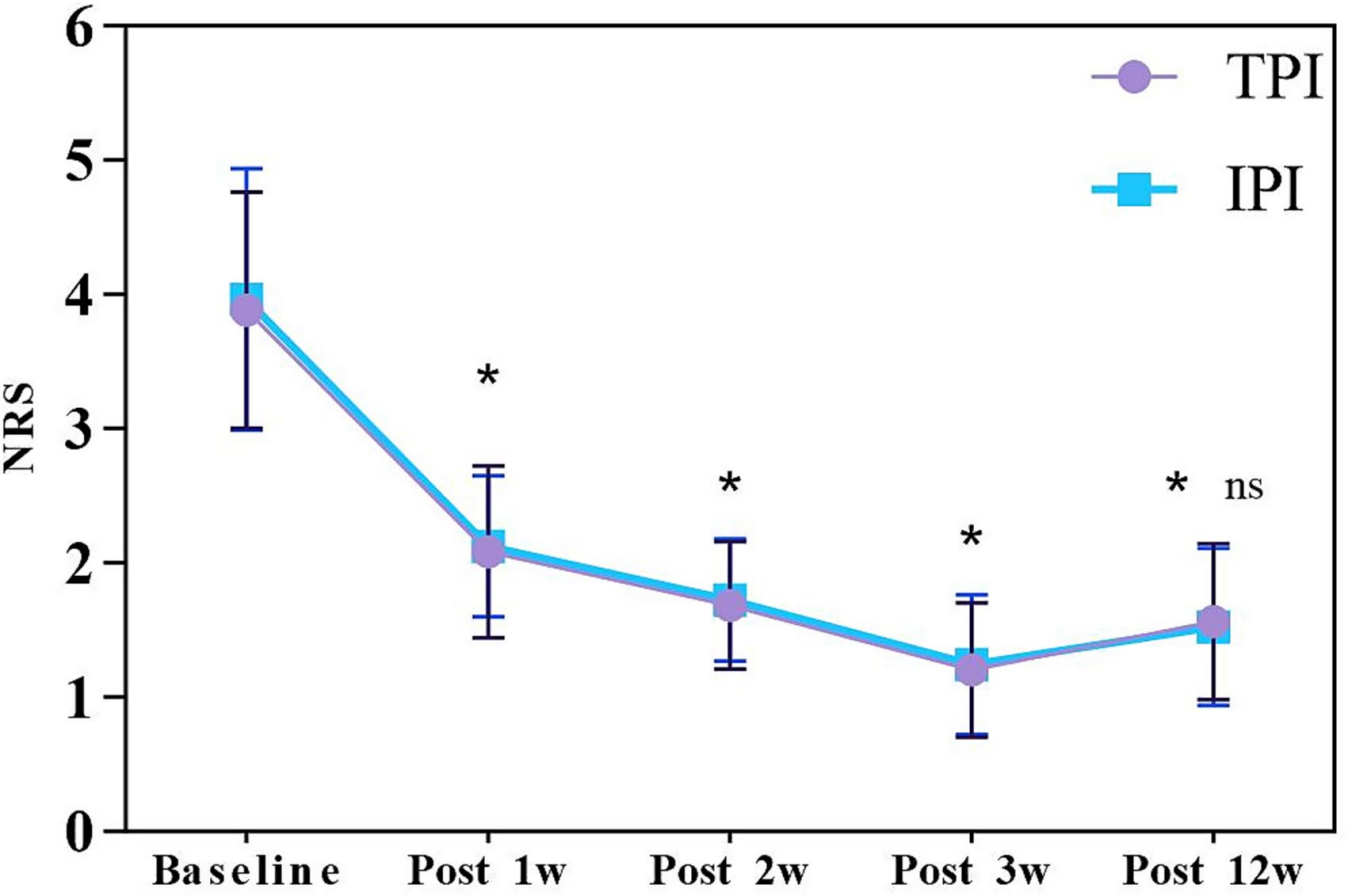
NRS changes in TPI and IPI groups. Data are presented as mean ± SD. * indicates *p* < 0.05 vs. baseline; ns indicates *p* > 0.05 for between-group comparison. NRS, Numeric Rating Scale; TPI, trigger point injection; IPI, interfascial plane injection.

Two-way repeated measures analysis of covariance (S), with baseline NRS scores as the covariate, revealed a statistically significant main effect of time on NRS scores (*F* (2.926, 137.532) = 4.630, *p* = 0.004; Greenhouse–Geisser corrected). The 95% CIs for the mean reduction in NRS scores from baseline to 12 weeks were [1.42, 1.98] for the TPI group and [1.38, 1.95] for the IPI group, both exceeding the minimum clinically important difference (MCID) of 1.5. In contrast, no significant main effect of group was observed (*F* (1, 47) = 0.245, *p* = 0.623), and there was no significant time-by-group interaction effect (*F* (2.926, 137.532) = 0.158, *p* = 0.921), with the 95% CIs for between-group differences at all time points crossing zero. This indicates that while both groups achieved marked pain relief, the magnitude and trend did not differ significantly between them. Post-hoc tests confirmed no statistically significant between-group differences at any time point (all *p* > 0.05) (Table 2; Figure 3).

**Table 2:** NRS changes in TPI and IPI groups.

Group	Baseline	Post 1 week	Post 2 week	Post 3 week	Post 12 week
TPI	3.88 ± 0.86	2.08 ± 0.63	1.68 ± 0.47	1.20 ± 0.49	1.56 ± 0.57
IPI	3.96 ± 1.01	2.12 ± 0.52	1.72 ± 0.45	1.24 ± 0.51	1.52 ± 0.57

TPI, trigger point injection; IPI, interfascial plane injection.

### 3.3 Pain quality (SF-MPQ)

**Figure 4:**
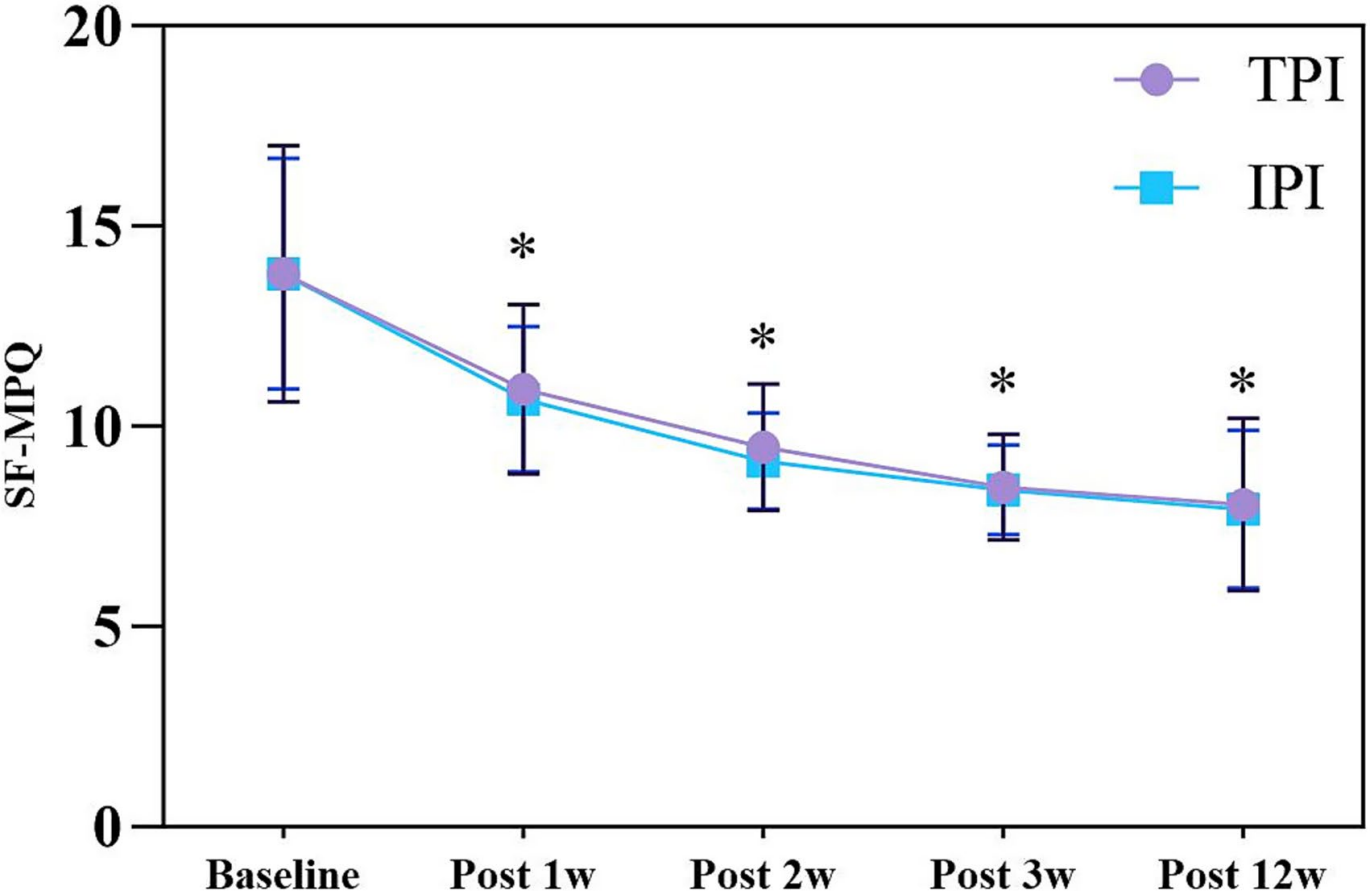
SF-MPQ changes in TPI and IPI groups. Data are presented as mean ± SD. * indicates *p* < 0.05 vs. baseline. SF-MPQ, Short-Form McGill Pain Questionnaire; TPI, trigger point injection; IPI, interfascial plane injection.

Two-way RM-ANCOVA (baseline NRS as covariate) demonstrated a statistically significant main effect of time on SF-MPQ scores (*F* (2.015, 94.689) = 7.710, *p* = 0.001; Greenhouse–Geisser corrected). The 95% CIs for the reduction in SF-MPQ scores indicated a consistent and substantial improvement in pain quality for both groups throughout the 12-week follow-up period. In contrast, no significant main effect of group was observed (*F* (1, 47) = 0.212, *p* = 0.647), and there was no significant time-by-group interaction effect (*F* (2.015, 94.689) = 0.093, *p* = 0.912; Greenhouse–Geisser corrected). The 95% CIs for the between-group differences in SF-MPQ scores at each time point consistently overlapped with zero, indicating that the analgesic efficacy and sensory-affective improvements were comparable between the IPI and TPI techniques. Post-hoc independent samples t-tests with Bonferroni correction confirmed that there were no statistically significant between-group differences in SF-MPQ scores at any post-treatment time point (all *p* > 0.05) (Figure 4).

### 3.4 Functional disability (NDI)

**Figure 5:**
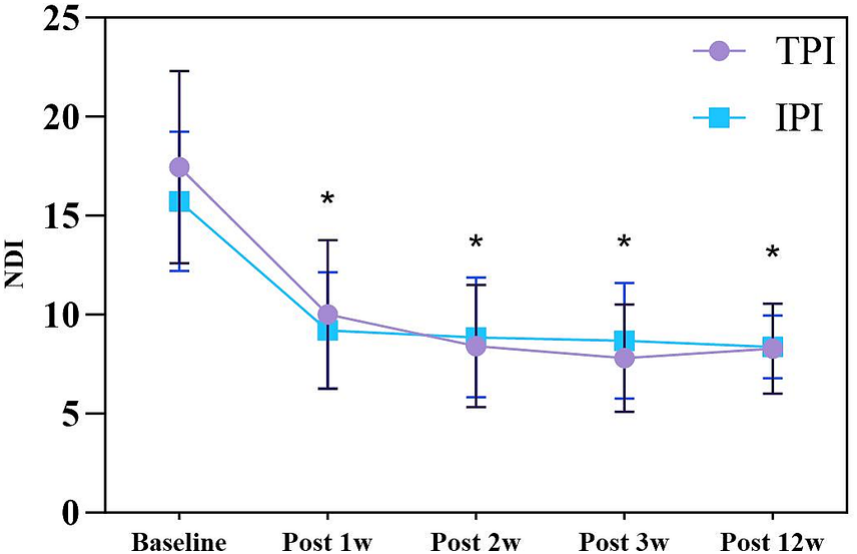
NDI changes in TPI and IPI groups. Data are presented as mean ± SD. * indicates *p* < 0.05 vs. baseline. NDI, Neck Disability Index; TPI, trigger point injection; IPI, interfascial plane injection.

Two-way RM-ANCOVA (baseline NRS as covariate) demonstrated a statistically significant main effect of time on NDI scores (*F* (2.717, 127.701) = 8.989, *p* < 0.001; Greenhouse–Geisser corrected). The 95% CIs for NDI improvement demonstrated a consistent downward trend in functional disability for both groups over the 12-week period. In contrast, no significant main effect of group was observed (*F* (1, 47) = 0.055, *p* = 0.816), and there was no significant time-by-group interaction effect (F (2.717, 127.701) = 2.499, *p* = 0.068). Post-hoc independent samples t-tests confirmed that there were no statistically significant between-group differences in NDI scores at any post-treatment time point (all *p* > 0.05) (Figure 5).

### 3.5 Muscle stiffness (shear-wave Elastography)

**Figure 6:**
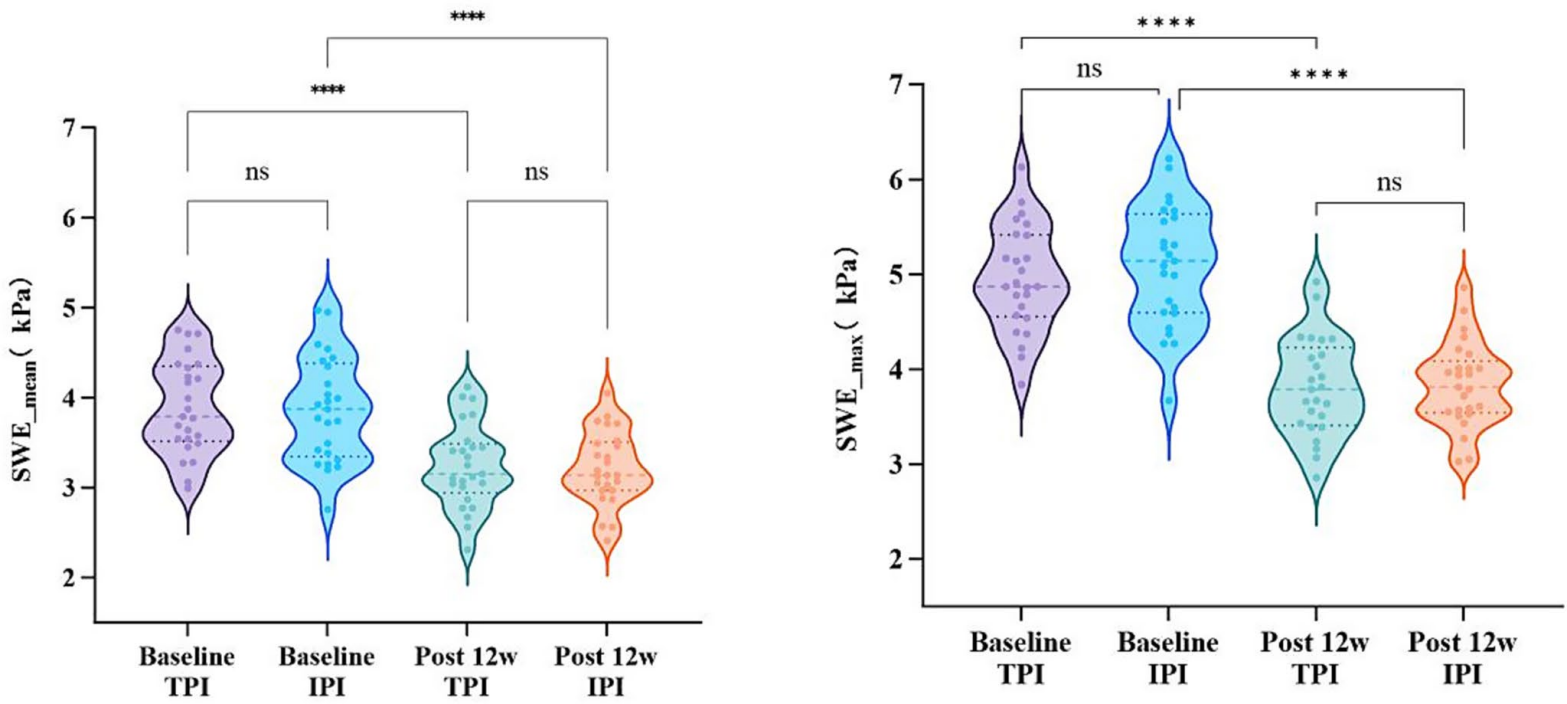
SWE__mean_ and SWE__max_ changes in TPI and IPI groups. Data are presented as mean ± SD. The violin plots illustrate the distribution of shear wave elastography (SWE) values. **** indicates *p* < 0.001 vs. baseline; ns indicates *p* > 0.05 for between-group comparison. SWE__mean_, Mean stiffness value measured by shear-wave elastography (kPa); SWE__max_, Maximum stiffness value measured by shear-wave elastography (kPa); TPI, trigger point injection; IPI, interfascial plane injection.

**Figure 7:**
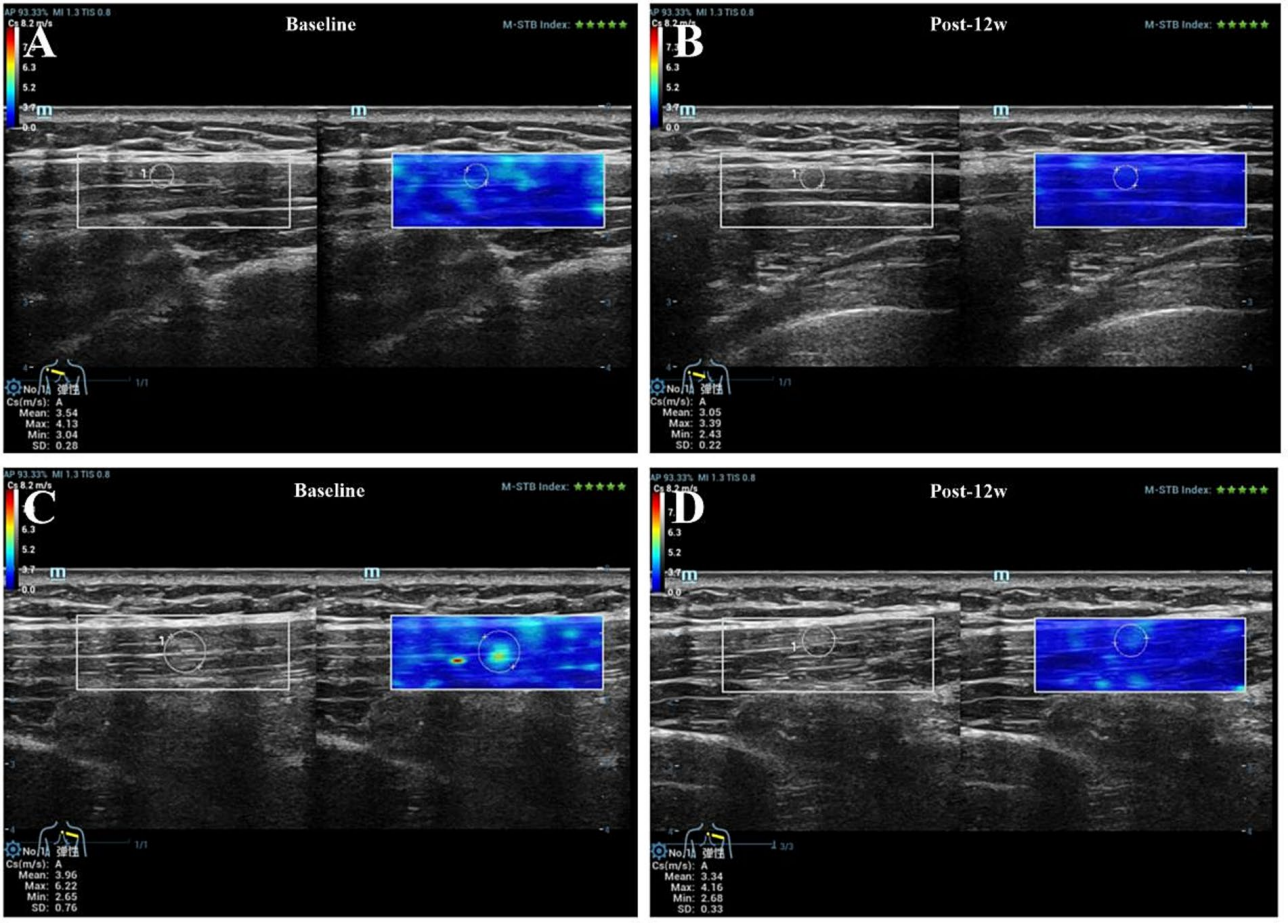
SWE of the upper trapezius muscle in 2 young female patients. Panels **A,B** were from a patient in the TPI group, and Panels **C,D** were from a patient in the IPI group, with assessments conducted before treatment and at 12 weeks post-treatment, respectively. For the TPI group: SWE__mean_ = 3.54 kPa, SWE__max_ = 4.13 kPa before treatment; SWE__mean_ = 3.05 kPa, SWE__max_ = 3.39 kPa at 12 weeks post-treatment. For the IPI group: SWE__mean_ = 3.96 kPa, SWE__max_ = 6.22 kPa before treatment; SWE__mean_ = 3.34 kPa, SWE__max_ = 4.16 kPa at 12 weeks post-treatment. SWE__mean_, Mean stiffness value measured by shear-wave elastography (kPa); SWE__max_, Maximum stiffness value measured by shear-wave elastography (kPa).

Two-way RM-ANCOVA (baseline NRS as covariate) demonstrated a statistically significant main effect of time for both SWE__mean_ and SWE__max_ values (both *p* < 0.001), indicating a marked reduction in muscle stiffness at 12 weeks post-treatment in both groups. No significant main effects of group or time-by-group interactions were observed for SWE__mean_ or SWE__max_ (all *p* > 0.05), confirming comparable therapeutic responses in muscle stiffness improvement between the two groups (Table 3; Figures 6, 7).

**Table 3:** SWE__mean_ and SWE__max_ changes in TPI and IPI groups.

Variable	Group	Baseline Mean ± SD	Post 12 week Mean ± SD	Mean Change [95% CI]	Time effect *p*	Group effect *p*
SWE__mean_ (kPa)	TPI	3.89 ± 0.52	3.24 ± 0.47	−0.65 [−0.78, −0.52]	< 0.001	0.866
	IPI	3.87 ± 0.58	3.21 ± 0.41	−0.66 [−0.79, −0.53]		
SWE__max_ (kPa)	TPI	4.95 ± 0.55	3.81 ± 0.52	−1.14 [−1.32, −0.96]	< 0.001	0.532
	IPI	5.09 ± 0.64	3.83 ± 0.45	−1.26 [−1.45, −1.07]		

SWE__mean_, Mean stiffness value measured by shear-wave elastography (kPa); SWE__max_, Maximum stiffness value measured by shear-wave elastography (kPa).

### 3.6 Adverse events

Over the course of 150 injection sessions (75 in each group), the TPI group reported 8 adverse events (incidence 10.7%; 95% CI 4.7–19.7%), including 6 cases of injection pain and 2 cases of skin ecchymosis. The IPI group reported only 1 adverse event (incidence 1.3%; 95% CI 0.03–7.2%), consisting of a single case of injection pain. No serious adverse events were recorded. The difference in adverse event rates between groups was statistically significant (*p* = 0.034).

### 3.7 Patient satisfaction

**Figure 8:**
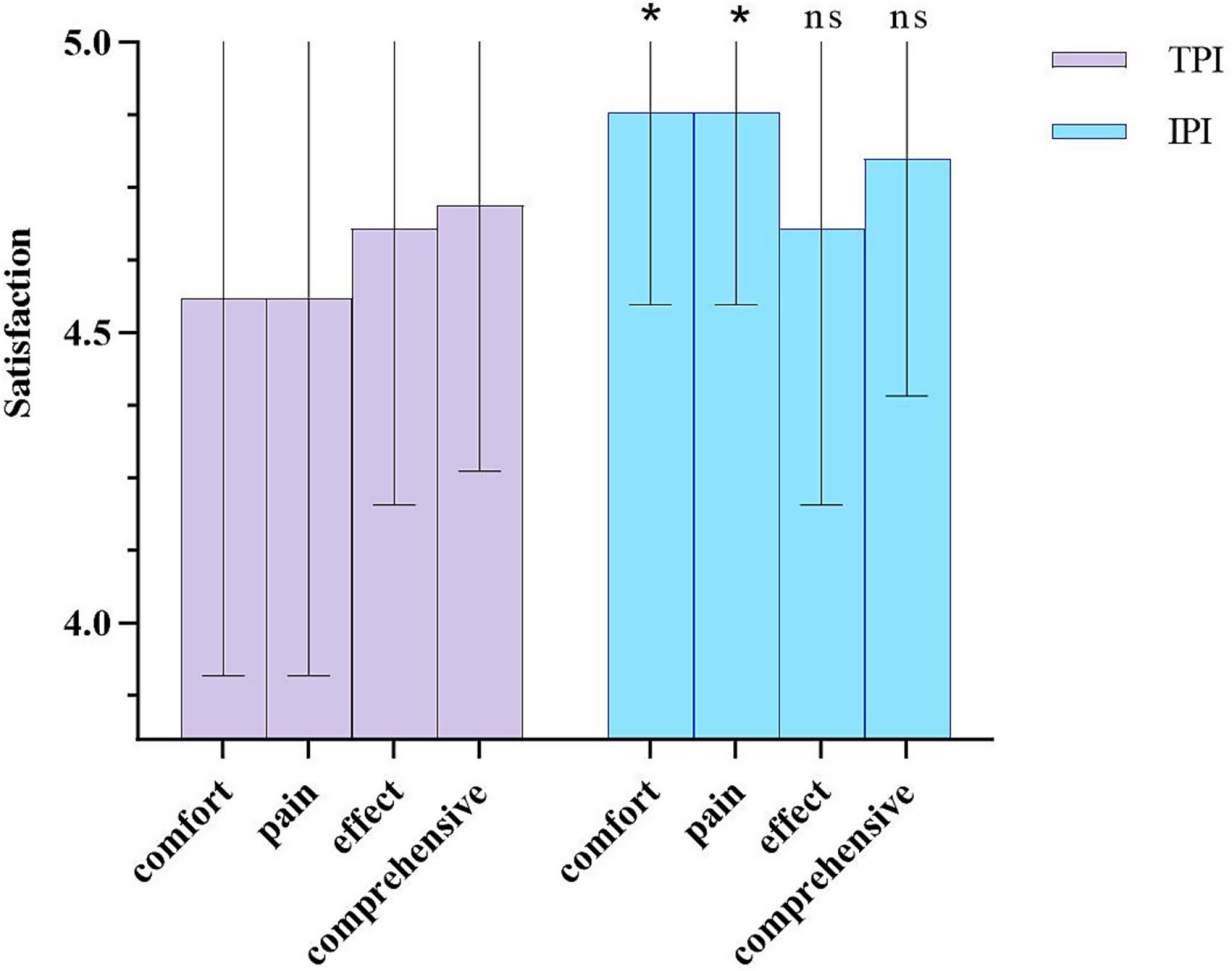
Patient satisfaction scores in the TPI and IPI groups. Data are presented as mean ± SD. * indicates *p* < 0.05 vs. TPI group; ns indicates *p* > 0.05 vs. TPI group. TPI, trigger point injection; IPI, interfascial plane injection.

Patient satisfaction was assessed using a self-designed questionnaire with good internal consistency (Cronbach’s *α* = 0.787), the questionnaire covering four domains: treatment comfort, pain tolerance, efficacy satisfaction, and overall satisfaction. Patients in the IPI group reported significantly higher scores for treatment comfort and pain tolerance compared to the TPI group (both *p* = 0.033). No significant between-group differences were found for efficacy satisfaction or overall satisfaction (Figure 8).

## 4 Discussions

The present study demonstrates that both IPI and TPI are effective in alleviating pain, improving function, and reducing muscle stiffness in young women with UT-MPS over a 12-week period. Notably, while the two modalities did not differ significantly in terms of therapeutic efficacy, IPI was associated with fewer adverse events and higher patient satisfaction regarding procedural comfort and pain tolerance. These findings suggest that IPI may serve as a viable and patient-friendly alternative to TPI, especially in clinical settings where resource constraints or operator experience limit the feasibility of precision trigger point localization (20).

Previous studies have confirmed that deactivating trigger points and releasing fascia can effectively alleviate pain and functional impairment in patients with MPS. Lew conducted a meta-analysis using a random-effects model to compare manual therapy and dry needling for treating myofascial pain syndrome in the neck and back. The study included six randomized controlled trials involving 241 patients. Results indicated that both treatment methods improved pain and limb function in patients in the short to medium term (21). For patients with chronic conditions, single-modality physical therapy often fails to achieve optimal results. TPI, which combine mechanical release with the relaxing effects of injected medications, represent the most widely used injection therapy currently available. It may provide better short-term pain relief than conservative treatments such as dry needling (22). There is currently no unified standard for selecting injectable medications, and there is a lack of high-quality evidence to validate whether there are significant differences in efficacy among different drugs (23). Two approaches are commonly employed for TPI: the first involves repeated needle stimulation of the trigger point prior to the slow injection of medication, and the second entails injecting medications upon needle placement followed by massage to facilitate drug diffusion into the surrounding trigger point region (24). In young female patients, local anesthetics are preferred to relieve injection pain, and the latter technique is adopted to minimize painful sensations during the procedure. Persistent pain or suboptimal treatment outcomes in patients with MPS are also closely related to the fascial system (25). Long-term chronic inflammatory responses and metabolic abnormalities can lead to excessive hyaluronic acid production, resulting in dense adhesions within the local fascia. This impairs the fascia’s normal elasticity and gliding function (26). Zhao demonstrated that sliding imbalance between the deep fascia and muscular layers may represent a potential pathophysiological mechanism underlying myofascial pain and dysfunction (27). Chen employed ultrasound-guided myofascial hydrodissection (UMHT) to restore normal gliding function within the fascial system by leveraging the diffusion tension of saline solution. Research findings indicate no statistically significant difference in core efficacy between this approach and trigger point injection (1% lidocaine injection) (19). Suarez-Ramos compared the efficacy of 2% lidocaine solution interfascial plane injection with dry needling for treating UT-MPS, further validating the effectiveness and clinical value of interfascial injection (28). The findings of this study are consistent with those of previous research, indicating that both TPI and IPI are effective therapeutic modalities for UT-MPS. No statistically significant difference in therapeutic efficacy was observed over a 12-week follow-up period. IPI may thus serve as a viable alternative therapy for UT-MPS. Notably, both groups had a mild rebound in NRS scores at the 12-week follow-up, which may be linked to young women’s prolonged head-down posture in work and leisure.

UT-MPS also involves pathological changes such as collagen deposition and inflammatory infiltration, which collectively contribute to increased tissue stiffness (29). SWE enables the quantitative assessment of the elastic stiffness response of muscle to external forces, translating its mechanical properties into visual images and quantitative parameters (30). This provides a valuable objective reference for disease evaluation (31). Hao demonstrated via evaluations of patients with UT-MPS and healthy volunteers that SWE can quantitatively reflect trapezius muscle stiffness. The elastic stiffness on both sides (affected and unaffected) in patients exhibited significantly higher stiffness compared with healthy individuals (32). Additionally, Valera found no significant difference in elastic stiffness between activated and dormant trigger points, indicating that SWE exhibits a nonlinear relationship with clinical severity (33). Anwar administered extracorporeal shock wave therapy combined with TPI to patients with UT-MPS. The results demonstrated significant pain reduction post-treatment, alongside decreased muscle elastic stiffness (34). This study also measured SWE of the upper trapezius muscle. The findings were consistent with previous research, indicating that TPI and IPI improved patient symptoms while concurrently reducing muscle stiffness.

The primary distinction from previous studies lies in its focus on the treatment experience of young female patients. Results indicate that in terms of adverse events, the incidence of injection pain was significantly lower in the IPI group compared with the TPI group. Regarding patient satisfaction, scores for both treatment comfort and pain tolerance were higher in the IPI group than in the TPI group. These findings underscore the clinical value of IPI in the young female patient population. The local twitch response induced by TPI is significantly correlated with therapeutic efficacy (35). To maximize treatment benefits, some clinicians often employ strategies involving stimulation of multiple trigger points or repeated stimulation to induce muscle twitch responses. Such practices inevitably cause iatrogenic tissue damage, leading to adverse reactions such as localized soreness and pain (36). This type of injury is more pronounced in patients with low tolerance for needle pain. Safety remains a core principle in healthcare services, while comfort enhancement represents a key trend in medical development. The low adverse event rate and high comfort level of IPI better align with the medical needs of young female patients.

In addition to its therapeutic efficacy and safety advantages, IPI features simple operation and low equipment dependency. By contrast, TPI requires precise trigger point localization, which demands a high level of operator proficiency and advanced ultrasound equipment, thus limiting its implementation in primary care settings. IPI, however, only requires the identification of fascial gaps and does not rely on high-resolution ultrasound devices, it offer practical advantages in clinical settings where procedural simplicity is prioritized. UT-MPS is an extremely prevalent painful condition in pain management, and primary care facilities serve as the main providers of its diagnosis and treatment (37). The ability to deliver safe, effective, and highly satisfactory treatment options for this condition—even under resource-constrained conditions—carries significant public health implications.

This prospective cohort study compared two established injection techniques routinely used for UT-MPS. Although both approaches provide effective symptomatic relief, they do not directly address the multifactorial etiology of UT-MPS (e.g., ergonomic strain, muscle imbalance, and psychosocial stressors). Injection therapy should be viewed as a “bridge” treatment—by rapidly alleviating severe pain and muscle stiffness, it creates a “therapeutic window” that allows patients to better tolerate and engage in subsequent long-term interventions, such as postural correction and physical rehabilitation (38). While the non-randomized design may introduce selection bias, we adopted a pragmatic clinical approach to reflect real-world practice, where patient preference and physician expertise are integral to treatment adherence. To minimize systematic differences, we implemented standardized eligibility criteria and blinded outcome assessment. Furthermore, we employed repeated-measures ANCOVA with baseline NRS scores as a covariate to statistically adjust for initial group differences and potential confounding. While baseline characteristics were comparable, supporting valid comparisons, residual confounding from unmeasured factors cannot be fully excluded.

First, this study utilized a pragmatic prospective cohort design rather than a randomized controlled trial. While this approach reflects real-world clinical decision-making and patient preference, it inherently introduces a risk of selection bias and unmeasured confounding. Although baseline characteristics were comparable and statistical adjustments (RM-ANCOVA) were employed, the sample size was relatively small and restricted to young women, which may limit generalizability to other demographic groups. The study was powered to detect clinically meaningful differences (MCID = 1.5), yet future multicenter randomized trials with larger, more diverse cohorts are needed to validate these findings.

## 5 Conclusion

This study compared IPI and TPI for UT-MPS in young women; both techniques demonstrated comparable and significant longitudinal improvements in pain intensity, functional disability, and muscle stiffness throughout the 12-week follow-up period. While clinical efficacy was equivalent, IPI was associated with a significantly lower incidence of adverse events and superior patient-reported treatment comfort and satisfaction. Given its comparable efficacy and favorable safety profile, IPI may offer practical advantages in clinical settings where procedural simplicity is prioritized.

## Data Availability

The raw data supporting the conclusions of this article will be made available by the authors, without undue reservation.
