# Supplementary material for: Comparison of interfascial plane injection and trigger point injection for upper trapezius myofascial pain syndrome in young women: a prospective cohort study
Source: Front Med (Lausanne). 2026 May 11;13:1819707. doi: 10.3389/fmed.2026.1819707 (PMC13199076; doi:10.3389/fmed.2026.1819707)
Supplement: Supplementary file 1 [file Table_1.doc]

Participant Information Page

Dear Participant,

You are invited to participate in the clinical research project entitled —A Comparison of Interfascial Plane Injection versus Trigger Point Injection for Upper Trapezius Myofascial Pain Syndrome in Young Women: A Prospective Cohort Study, which is supported by **Ningbo Yinzhou District No.2 Hospital**. Please read this informed consent form carefully and make a prudent decision on whether to participate in this study.

1. **Your Core Rights**
   Your participation in this study is entirely your voluntary choice. As a participant, you must provide your written informed consent before joining the study. If you encounter any content you do not understand during discussions with your research doctor or other researchers about the informed consent form, you may request an explanation. We encourage you to have thorough discussions with your family and friends before making a decision to participate in this study. You have the right to refuse to participate in this study or withdraw at any time without penalty or loss of any rights you are entitled to. If you are currently participating in another study, please inform your research doctor or researcher.
2. **Research Purpose**
   Upper trapezius myofascial pain syndrome is a musculoskeletal disorder with a high incidence among young women, often leading to neck and shoulder pain, stiffness, and cervical dysfunction, severely impacting quality of life. Ultrasound-guided trigger point injection is a standard clinical treatment; however, it demands advanced technical skills and equipment, and some patients may experience discomfort during the procedure. In contrast, interfascial plane injection is simpler to perform and offers broader coverage, yet no targeted research has compared its therapeutic efficacy with that of trigger point injection.
   This study aims to compare the analgesic effects, improvement in cervical function, and changes in muscle stiffness between the two ultrasound-guided injection therapies, while also assessing treatment safety and patient comfort. The goal is to validate the clinical value of interfascial plane injection and provide a superior treatment option for young women with this condition, particularly for clinical application in primary care settings.
3. **Subject Inclusion Criteria**
   Inclusion Criteria:
   (1) Female, aged 18–44 years, with a body mass index (BMI) of 18.5–24 kg/m²;
   (2) Meeting the clinical diagnostic criteria for upper trapezius myofascial pain syndrome: unilateral neck and shoulder pain persisting for ≥3 months, baseline Numeric Pain Rating Scale (NRS) score ≥3, Neck Disability Index (NDI) score >8, presence of an active trigger point palpable in the upper trapezius muscle with reproduction of referred pain upon pressure;
   (3) No receipt of physical therapies such as acupuncture or needle-knife treatment within the past 3 months, and no use of glucocorticoid therapy within the past 1 month;
   (4) Ability to cooperate with the treatment and complete the 12-week follow-up assessments. Exclusion Criteria:
   (1) Presence of other organic neck and shoulder diseases, such as cervical disc herniation or periarthritis of the shoulder;
   (2) Presence of severe cardiovascular, cerebrovascular, neurological, or autoimmune diseases, or severe gastrointestinal diseases (contraindication to nonsteroidal anti-inflammatory drugs);
   (3) Skin damage or infection in the treatment area, or coagulation dysfunction/bleeding tendency;
   (4) Allergy to local anesthetics (e.g., ropivacaine);
   (5) Pregnancy or lactation;
   (6) Poor compliance, inability to cooperate with treatment and follow-up.
4. **Study Process and Schedule**
   If you meet the inclusion criteria and agree to participate in the study, the entire process will be completed at the Department of Pain Medicine, Ningbo Yinzhou District Second Hospital. All study-related examinations and treatments will be provided free of charge, and you will not incur any costs. The specific procedures are as follows:
5. Baseline Assessment (Pre-treatment): Complete a physical examination, along with assessments including the Numeric Pain Rating Scale (NRS), Neck Disability Index (NDI), Short-Form McGill Pain Questionnaire (SF-MPQ), and ultrasound shear wave elastography (SWE) to evaluate upper trapezius muscle stiffness. All examinations will be performed by professional physicians.
6. Treatment Protocol: Based on your preference and the clinical judgment of the study physician, you will be assigned to either the interfascial plane injection (IPI) group or the trigger point injection (TPI) group. Both groups will receive ultrasound-guided injections using a 22G needle with 0.2% ropivacaine. Treatment will be administered once weekly for three sessions, with each session lasting approximately 10–15 minutes. During treatment, you will be seated, and only the neck and shoulder area needs to be exposed.
7. Post-Treatment Follow-Up Assessments: Follow-up assessments will be conducted at weeks 1, 2, 3, and 12 after treatment. At weeks 1, 2, and 3, the NRS, SF-MPQ, and NDI will be evaluated. At week 12, in addition to the above assessments, an additional ultrasound shear wave elastography (SWE) examination will be performed. All follow-ups will take place at the outpatient clinic, with each session lasting approximately 15–20 minutes.
8. Safety and Satisfaction Evaluation: Any adverse reactions will be recorded after each treatment session. At the week 12 follow-up, you will complete a patient satisfaction questionnaire (assessing treatment comfort, pain tolerance, etc.), which will take approximately 5 minutes.

Your total participation period in the study is 12 weeks, during which you will complete one baseline assessment, three treatment sessions, and four follow-up visits, with no additional time commitment required.

1. **Risks and Benefits**

Risks of Participating in This Study: Both injection therapies used in this study are routine, well-established clinical procedures performed under ultrasound guidance. The administered 0.2% ropivacaine is a low-concentration local anesthetic with a high safety profile and no risk of severe adverse reactions. Any mild discomfort that may occur is transient and can resolve spontaneously or with simple management, including:

1. Temporary soreness or mild pain at the injection site, typically resolving within a few hours;
2. In rare cases, minor skin bruising at the injection site, which usually fades within 1–2 weeks;
3. In the event of an allergic reaction to the local anesthetic (extremely low incidence), the study physician will immediately administer antiallergic treatment to ensure your safety.
4. If you experience any discomfort during the study, you should promptly inform the study physician, and we will provide timely medical attention.

Benefits of Participating in This Study:

1. Receive standardized ultrasound-guided injection therapy free of charge, along with all study-related examinations, reducing medical expenses;
2. Targeted treatment for neck and shoulder pain, stiffness, and other symptoms, effectively improving cervical function and enhancing quality of life;
3. Access to continuous follow-up guidance from professional physicians, enabling timely understanding of changes in your condition.
4. **Alternative Treatment Options (Are there other current treatment methods?)**

Pharmacological therapy generally has minimal therapeutic effects.

1. **Use of Research Results and Confidentiality of Personal Information**

If you decide to participate in this study, your involvement and personal data collected during the trial will be kept strictly confidential. Information that could identify you will not be disclosed to individuals outside the research team unless we obtain your permission. All research team members and the research sponsor are required to protect your identity. Your records will be stored in locked filing cabinets accessible only to the research personnel. To ensure the study is conducted properly, authorized representatives from government regulatory authorities or the Ethics Committee may, when necessary, review your personal data at the study site in accordance with applicable regulations. If the results of this study are published, no personally identifiable information will be disclosed.

1. **Other Study-Related Information**

**Study Costs:** This study is a non-profit clinical research project. All study-related examinations, treatments, and medications are provided free of charge, and you will not incur any additional medical expenses. If you develop conditions unrelated to the study during the research period, the treatment costs for such conditions will be your responsibility.

**Confidentiality of Personal Information:** Your personal information, including name, ID number, and contact details, will be stored separately and kept strictly confidential. Study data will be recorded using only identification numbers and will be accessible solely to research team members. If regulatory authorities such as the Ethics Committee or drug administration departments require audits, they will conduct them in strict compliance with relevant regulations and will not disclose your identity information.

**Use of Research Results:** The results of this study will be used for academic publication and clinical research reports. Only aggregated statistical data will be presented, and no information that could identify you will be disclosed.

1. **Participant Responsibilities**

As a participant, you are required to provide truthful information about your medical history and current physical condition. You should inform the study physician of any discomfort you experience during the study period. You must not take any restricted medications or consume any restricted foods as advised by the physician. You should also inform the study physician if you have recently participated in or are currently participating in other studies.

1. **Contact Information for Further Information**

If any important new information arises during the study that may affect your willingness to continue participating, your physician will notify you promptly. If you have questions about your study data or wish to learn about the findings of the study after its conclusion, you may raise any questions regarding this study at any time and receive corresponding answers. Please contact Jiang Ren at 13989309561.

This study has been reviewed and approved by the Ethics Committee. If you have any questions related to your rights or interests, wish to report difficulties, dissatisfaction, or concerns encountered during your participation, or would like to provide suggestions or opinions regarding this study, please contact our hospital's Ethics Committee.

**Subject Signature Page**

Informed Consent Statement:

I have been informed of the purpose, background, procedures, risks, and benefits of this study. I have had sufficient time and opportunity to ask questions, and I am satisfied with the answers provided.

I have also been informed of whom to contact if I have questions, wish to report difficulties, concerns, or suggestions regarding the study, need further information, or would like to offer assistance with the research.

I have read this informed consent form and agree to participate in this study.

I understand that I may choose not to participate in this study or withdraw from it at any time during the study period without giving any reason.

I understand that if my condition worsens, if I experience a serious adverse event, or if my study physician believes that continuing participation is not in my best interest, he/she may decide to withdraw me from the study. The sponsor or regulatory authorities may also terminate the study during its course without requiring my consent. If such a situation occurs, the physician will notify me promptly and discuss other options with me.

I will receive a copy of this informed consent form, containing the signatures of both myself and the researcher.

Finally, I confirm that I have understood all of the above and agree to participate in this clinical research project.

The subject confirms understanding of the above content (to be handwritten):

Subject Signature：_________________________ Date：________________________

(Note: If the subject lacks capacity or has limited capacity, a signature and date from the legal guardian are required.)

Subject Contact Information: _____________________________

Legal Guardian Signature: _______________________ Date: __________________________

Legal Guardian Contact Information: ______________________________

Investigator Signature: _________________________ Date: __________________________

Investigator Contact Information: ______________________________
