# Supplementary material for: Comparison of interfascial plane injection and trigger point injection for upper trapezius myofascial pain syndrome in young women: a prospective cohort study
Source: Front Med (Lausanne). 2026 May 11;13:1819707. doi: 10.3389/fmed.2026.1819707 (PMC13199076; doi:10.3389/fmed.2026.1819707)
Supplement: Supplementary file 2 [file Table_2.doc]

受试者须知页

**尊敬的受试者：**

您被邀请参加“筋膜间隙注射与触发点注射治疗年轻女性上斜方肌肌筋膜疼痛综合征的前瞻性队列研究”，该项研究由宁波市鄞州区第二医院提供支持。请仔细阅读本知情同意书并慎重作出是否参加本项研究的决定。

1. **您的核心权利**

参加这项研究完全是您自主的选择。作为受试者，您必须在加入研究前给出您的书面同意书。当您的研究医生或其它研究人员和您讨论知情同意书的时候，您可以要求解释您看不明白的地方。我们鼓励您在作出参与此项研究的决定之前，和您的家人及朋友进行充分的讨论。您有权拒绝参加本研究，也可随时退出研究，且不会受到处罚，也不会失去您应有的权利。若您正在参加别的研究，请告诉您的研究医生或研究人员。

1. **研究目的**

上斜方肌肌筋膜疼痛综合征是年轻女性高发的肌肉骨骼疾病，常导致颈肩部疼痛、僵硬、颈椎功能障碍，严重影响生活质量。超声引导下触发点注射是临床常规治疗方法，但对操作技术和设备要求较高，且部分患者会出现注射不适；筋膜间隙注射操作更简便、覆盖范围更广，但其与触发点注射的疗效对比尚无针对性研究。

本研究通过对比两种超声引导下注射疗法的镇痛效果、颈椎功能改善情况、肌肉硬度变化，同时评估治疗的安全性和患者舒适度，验证筋膜间隙注射的临床价值，为年轻女性该疾病的临床治疗，尤其是基层医院的临床应用，提供更优选择。

1. **研究对象纳入要求**

（一）纳入标准：

1. 年龄 18-44 周岁，女性，体重指数（BMI）18.5-24kg/m²；
2. 符合上斜方肌肌筋膜疼痛综合征临床诊断标准：单侧颈肩部疼痛持续≥3个月，基线数字疼痛评分（NRS）≥3分，颈椎功能障碍指数（NDI＞8分，上斜方肌可触及活动性触发点，按压可复制牵涉痛；
3. 3个月内未接受针灸、小针刀等物理治疗，1个月内未使用糖皮质激素治疗；
4. 能够配合完成治疗及为期 12 周的随访评估。
5. 排除标准：
6. 合并颈椎间盘突出、肩周炎等其他颈肩部器质性疾病；
7. 合并严重心脑血管、神经系统、自身免疫性疾病，或有严重胃肠道疾病（无法耐受非甾体抗炎药）；
8. 治疗区域皮肤破损、感染，或有凝血功能障碍、出血倾向；
9. 对局部麻醉药（罗哌卡因）过敏；
10. 妊娠、哺乳期女性；
11. 依从性差，无法配合治疗和随访者。
12. **研究过程与时间安排**

若您符合纳入标准并同意参加研究，研究过程将全程在宁波市鄞州区第二医院疼痛科完成，所有研究相关的检查、治疗均为免费，您无需支付任何费用，具体流程如下：

1. 基线评估（治疗前）：完成一次体格检查，以及疼痛评分（NRS）、颈椎功能评估（NDI）、简版麦吉尔疼痛问卷（SF-MPQ）和超声剪切波弹性成像（SWE）检查（评估上斜方肌肌肉硬度），所有检查均由专业医师操作。
2. 治疗方案：根据您的意愿和研究医生的临床判断，您将被分配至筋膜间隙注射组（IPI） 或触发点注射组（TPI），两组均采用超声实时引导，使用 22G 注射针，注射 0.2% 罗哌卡因，每周治疗 1 次，共治疗 3 次，每次治疗时间约 10-15 分钟，治疗时取坐位，暴露颈肩部即可。
3. 术后随访评估：治疗后第 1、2、3、12 周各完成一次随访，其中第 1、2、3 周完成 NRS、SF-MPQ、NDI 评估，第 12 周除上述评估外，额外完成一次超声剪切波弹性成像检查；所有随访均为门诊完成，每次评估时间约 15-20 分钟。
4. 安全性与满意度评估：每次治疗后记录是否有不适反应，第 12 周随访时完成一份患者满意度问卷调查（评估治疗舒适度、疼痛耐受性等），问卷填写约 5 分钟。

您的整体研究参与周期为 12 周，全程需完成 1 次基线评估 + 3 次治疗 + 4 次随访，无额外时间占用。

1. **风险与收益**

**参加本研究的风险**：本研究采用的两种注射疗法均为临床常规、成熟的超声引导操作，使用的 0.2% 罗哌卡因为低浓度局部麻醉药，安全性高，无严重不良反应风险，可能出现的轻微不适均为一过性，可自行缓解或经简单处理后恢复，包括：注射部位短暂的酸胀、轻微疼痛，一般数小时内消失；极少数情况下可能出现注射部位皮肤轻微瘀斑，1-2 周内可自行消退；若对局部麻醉药过敏（发生率极低），研究医生会立即采取抗过敏治疗，确保您的安全。研究过程中若您出现任何不适，可立即告知研究医生，我们将为您提供及时的医疗处理。

**参加本研究受益**： 免费获得超声引导下的规范化注射治疗，以及研究期间的所有相关检查，节省医疗费用；针对性治疗颈肩部疼痛、僵硬等症状，有效改善颈椎功能，提升生活质量；获得专业医生的全程随访指导，及时了解自身病情变化。

**6.替代方案（目前是否有其它的治疗方法）**

药物治疗，一般药物治疗效果不明显。

**7.研究结果的使用和个人信息的保密**

如果您决定参加本项研究，您参加试验及在试验中的个人资料均属保密。可以识别您身份的信息将不会透露给研究小组以外的成员，除非获得您的许可。所有的研究成员和研究申办方都被要求对您的身份保密。您的档案将保存在有锁的档案柜中，仅供研究人员查阅。为确保研究按照规定进行，必要时，政府管理部门或伦理审查委员会的成员按规定可以在研究单位查阅您的个人资料。这项研究结果发表时，将不会披露您个人的任何资料。

1. **研究相关的其他说明**

**研究费用：**本研究为公益性临床研究，所有研究相关的检查、治疗、药物均免费，不会增加您的任何医疗费用；若研究过程中您出现非研究相关的疾病，治疗费用由您自行承担。

**个人信息保密：**您的姓名、身份证号、联系方式等个人信息将单独存档，严格保密；研究数据仅以编号形式录入，仅研究团队成员可查阅，伦理委员会、药品监督管理部门等监管机构如需核查，将严格按照相关规定执行，且不会泄露您的身份信息。

**研究结果使用：**本研究的结果将用于学术论文发表、临床研究报告，仅展示汇总的统计数据，不会披露任何可识别您身份的信息。

**9.受试者相关注意事项：**

作为受试者，您需要提供有关自身病史和当前身体状况的真实情况，告诉研究医生自己在本次研究期间所发现的任何不适，不得服用医生已告知的受限制的药物、食物等，告诉研究医生自己最近是否参与其他研究，或目前正参与其它研究。

**10.获知信息的相关联系方式：**

如果在研究过程中有任何重要的新信息，可能影响您继续参加研究的意愿时，您的医生将会及时通知您。如果您对自己的研究数据或研究结束后您希望知道本研究的发现，您可以在任何时候提出有关本项研究的任何问题，并得到相应的解答，请通过电话13989309561与江仁 联系。

伦理委员会已经审核通过本研究，如果您有与自身权利/权益相关的任何问题，或者您想反映参与本研究过程中遭遇的困难、不满和忧虑，或者想提供与本研究有关的建议和意见，请联系我院伦理委员会。

受试者签字页

知情同意声明：

我已被告知此项研究的目的、背景、过程、风险及获益等情况，我有足够的时间和机会进行提问，问题的答复我很满意。

我也被告知，当我有问题、想反映困难、顾虑、对研究的建议，或想进一步获得信息，或为研究提供帮助时，应当与谁联系。

我已经阅读这份知情同意书，并且同意参与本研究。

我知道我可以选择不参加此项研究，或在研究期间的任何时候无需任何理由退出本研究。

我已知道如果我的状况更差了，或者我出现严重的不良事件，或者我的研究医生觉得继续参加研究不符合我的最佳利益，他/她会决定让我退出研究。无需征得我的同意，资助方或者监督机构也可能在研究期间终止研究。如果发生该情况，医生将及时通知我，研究医生也会与我讨论我的其他选择。

我将得到这份知情同意书的副本，上面包含我和研究者的签名。

最后，我确定已理解上述全部内容，决定同意参加本临床研究项目。

患方对上述内容理解并确认（亲笔书写）：

受试者签名：_________________________ 日期：________________________

(注：如果受试者无行为能力/限制行为能力时，则需法定代理人签名和签署日期)

受试者联系方式：_____________________________

法定代理人签字：_______________________ 日期：__________________________

法定代理人联系方式：______________________________

研究者签名：_________________________ 日期：__________________________

研究者联系方式：______________________________
